# Hesperidin Is a Promising Nutraceutical Compound in Counteracting the Progression of NAFLD In Vitro

**DOI:** 10.3390/ijms26135982

**Published:** 2025-06-21

**Authors:** Miriam Cofano, Ilenia Saponara, Valentina De Nunzio, Giuliano Pinto, Emanuela Aloisio Caruso, Matteo Centonze, Maria Notarnicola

**Affiliations:** Laboratory of Nutritional Biochemistry, National Institute of Gastroenterology IRCCS “Saverio de Bellis”, 70013 Castellana Grotte, Italy; miriam.cofano@irccsdebellis.it (M.C.); ilenia.saponara@irccsdebellis.it (I.S.); valentina.denunzio@irccsdebellis.it (V.D.N.); giuliano.pinto@irccsdebellis.it (G.P.); emanuela.caruso@irccsdebellis.it (E.A.C.); matteo.centonze@irccsdebellis.it (M.C.)

**Keywords:** NAFLD, steatosis, β-oxidation, DNL, fibrosis markers, SIRT1, PGC1α, LX-2, Hepa-RG

## Abstract

Non-alcoholic fatty liver disease (NAFLD) is characterized by an accumulation of fat in hepatocytes, and it may progress, under additional triggering factors, to non-alcoholic steatohepatitis (NASH). Effective strategies to counteract this progression are essential, especially considering that at the moment, there is a lack of approved pharmacological therapies. Our previous study showed that the daily consumption of Navelina oranges significantly reduced hepatic steatosis in patients with Metabolic Dysfunction-Associated Fatty Liver Disease (MAFLD). Starting with our previous study, here, we have investigated the molecular targets through which Hesperidin (HE), a citrus flavanone, is able to prevent the progression of NAFLD to NASH using an in vitro model. In Hepa-RG cells exposed to NAFLD Promoting Agents, HE reduced lipid droplet accumulation (~35%) and suppressed de novo lipogenesis, with decreased expression of FASN (0.62 ± 0.06 vs. 0.39 ± 0.03 at 100 µg/mL) and SCD1 (0.05 ± 0.001 vs. 0.03 ± 0.004 at 50 µg/mL). HE also enhanced fatty acid oxidation by increasing SIRT1 (0.73 ± 0.16 vs. 2.36 ± 0.10 at 50 µg/mL) and PGC1α (0.71 ± 0.03 vs. 0.89 ± 0.003 at 50 µg/mL). In LX-2 cells, HE downregulated COL1A1 (1.48 ± 0.10 vs. 0.90 ± 0.11 at 100 µg/mL) and α-SMA (1.21 ± 0.16 vs. 0.76 ± 0.07 at 75 µg/mL) and upregulated MMP3 (0.64 ± 0.05 vs. 0.98 ± 0.07) and MMP9 (0.99 ± 0.005 vs. 2.61 ± 0.16 at 100 µg/mL). In conclusion, HE may offer a promising approach for NAFLD/NASH prevention and treatment, demonstrating in vitro its potential to reduce hepatic steatosis and fibrosis.

## 1. Introduction

Non-alcoholic fatty liver disease (NAFLD) is the most common chronic liver disease worldwide, which occurs in the absence of significant alcohol consumption and other secondary causes (such as congenital errors of metabolism or viral infections). It is characterized by ectopic fat accumulation in more than 5% of hepatocytes and increased secretion of hepatokines [1,2,3].

In the liver, lipid accumulation leads to insulin resistance and hepatic necroinflammation, triggering the activation of Hepatic Stellate Cells (HSC), which in turn increases collagen matrix production [4,5,6]. This promotes the progression of liver damage, resulting in a more aggressive phenotype known as NASH in 20–30% of cases. NASH is associated with fibrosis or scarring of the liver, which can increase the risk of cirrhosis, in the end leading to the development of Hepatocellular Carcinoma (HCC).

The pathogenesis underlying NAFLD/NASH remains only partially understood, and currently, no effective pharmacological treatments are available [7,8,9]. To further elucidate the molecular mechanisms driving NAFLD, the development of in vitro and in vivo models is crucial. These models could also play a key role in identifying effective therapeutic targets and agents.

Animal models often have limitations in accurately reflecting the metabolic or transcriptomic features of humans [10]. Thus, it is crucial to develop simple, reliable in vitro models that mimic the main features of NAFLD, particularly focusing on lipid metabolism in hepatocytes and the progression of fibrosis.

Evidence exists that, in the early stages of NAFLD, lipid accumulation in the liver is typically associated with an imbalance between hepatic fat delivery, derived from circulating lipids and increased de novo lipogenesis (DNL), on one hand, and decreased lipid disposal, mainly through Free Fatty Acid (FFA) oxidation and lipoprotein secretion, on the other [11,12,13,14].

DNL is a process in which cells convert excess carbohydrates into fatty acids via acetyl-CoA. Acetyl-CoA is then converted into malonyl-CoA and subsequently into Triglycerides (TG) through the action of enzymes such as Acetyl-CoA Carboxylase (ACC), Fatty Acid Synthase (FASN), and Stearoyl-CoA Desaturase-1 (SCD1), which regulate fatty acid synthesis [15,16,17]. To date, the study of these lipogenic modulators, for example in primary human hepatocytes and/or hepatic cell lines, is gaining increasing relevance, as it allows for a better understanding of their impact on various aspects of lipid metabolism, particularly on DNL, TG accumulation, and β-oxidation [18,19,20].

A key player in the β-oxidation of fatty acids (FAs) and in the regulation of mitochondrial function is the Peroxisome Proliferator-Activated Receptor Gamma Coactivator 1 alpha (PGC1α). Many studies have shown how PGC1α can exert its actions when deacetylated by Silent Information Regulator 2 homolog 1 (SIRT1). It has been reported that SIRT1/PGC1α improves NAFLD by regulating autophagy, fibrosis, endoplasmic reticulum stress, and oxidative stress [21,22,23,24,25]. Tong Nie et al. demonstrated that fatty acid β-oxidation is promoted by activating the SIRT1/PGC1α pathway in mouse liver cells treated with a combination of Oleic Acid (OA) and Palmitic Acid (PA). Interestingly, this study established that HE attenuates hepatic fat accumulation by modulating fatty acid oxidation [21]. HE natural flavanone glycoside found in high concentrations in citrus fruits has been shown to have antifibrotic and anti-steatotic effects both in vivo and in vitro. In various studies on HSC, it has been shown that different concentrations of HE suppressed the expression of Alpha-Smooth Muscle Actin (α-SMA) and Collagen Type 1 Alpha 1 Chain (COL1A1), thereby preventing the progression of liver fibrosis and the deposition of Extracellular Matrix (ECM) elements [26,27].

Saponara et al. also demonstrated how HE not only reduced protein levels of α-SMA, COL1A1, and Collagen Type 1 Alpha 2 Chain (COL1A2) but also reduced phosphorylation of Small Mother Against decapentaplegic 2 and 3 (SMAD 2/3) involved in the pathway of activation of HSC by Transforming Growth Factor-Beta (TGF-β) (5 ng/mL), suggesting how polyphenol interferes in the pathway of the activation of the aforementioned cells [28]. Additionally, HE has been shown to exhibit antisteatotic effects both in vivo and in vitro. In a 2022 study, the flavonoid was shown to suppress cellular lipid accumulation in a hepatocellular carcinoma cell line (HepG2) by the oil red staining technique and downregulated the expression of ACC and FASN [15]. Based on these considerations, we hypothesize that Hesperidin may exert its protective effects against the progression of NAFLD to NASH by specifically modulating key molecular targets involved in both lipid accumulation and fibrogenesis. HE may downregulate enzymes responsible for DNL, such as FASN and SCD1, while enhancing the expression of oxidative regulators like SIRT1 and PGC1α, which are known to promote fatty acid oxidation. At the same time, we expect HE to reduce fibrotic responses by suppressing the expression of fibrosis-related markers, including α-SMA and COL1A1. Through this combined action on lipid and fibrotic pathways, HE may help to mitigate liver damage and slow down or prevent the transition from simple steatosis to steatohepatitis.

Considering these aspects, this study will primarily focus on investigating the role of HE in both fibrosis and hepatic steatosis in vitro, utilizing the Human Hepatic Stellate Cell Line (LX-2) and healthy human hepatocytes (Hepa-RG). Specifically, both cell lines were activated with a mixture including several key agents such as glucose, insulin, FFA, lipopolysaccharide (LPS), and TGF-β, which mimic the pathophysiological liver condition observed in NAFLD patients.

## 2. Results

### 2.1. Effects of Increasing HE Concentrations on the Viability of Hepa-RG and LX-2 Hepatic Cells

**Figure 1:**
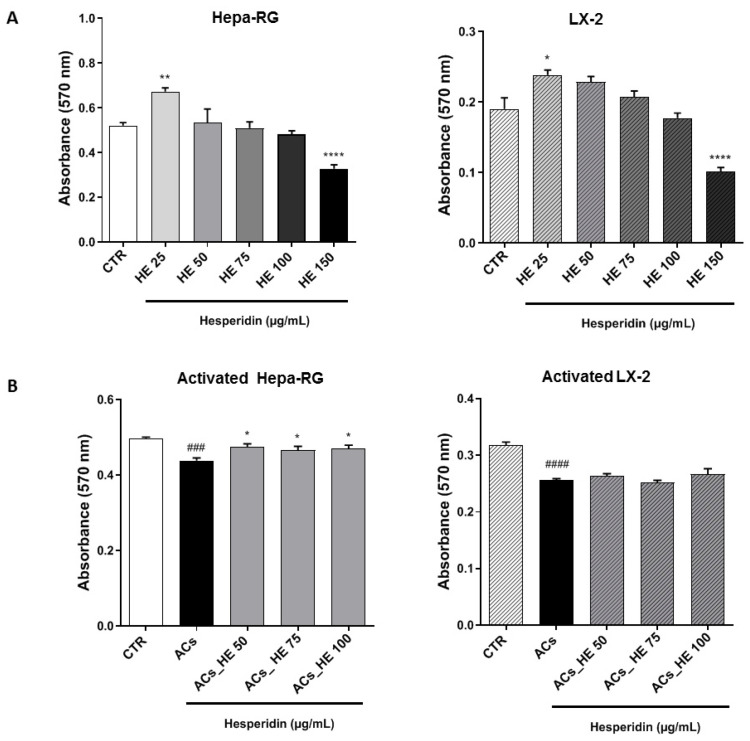
(**A**) Effects of HE on the cell viability of Hepa-RG and LX-2 cells and (**B**) activated Hepa-RG and LX-2 cells (ACs) treated with increasing concentrations of HE or its solvent (CTR) for 48 h. All data reported in each panel are expressed as the mean ± SEM from three independent experiments (n = 3 for each condition). Statistical analyses: one-way ANOVA Dunnet’s post-hoc analysis to compare differences between the control (CTR) and increasing concentrations of HE (25, 50, 75, 100, and 150 µg/mL) in (**A**) (* *p* < 0.05, ** *p* < 0.01, **** *p* < 0.0001). One-way ANOVA Dunnet’s post-hoc analysis to compare differences between CTR and activated cells (ACs), (### *p* < 0.001, #### *p* < 0.0001); statistical differences between activated cells (ACs) and increasing concentrations of HE (* *p* < 0.05) in (**B**).

The cytotoxic effects of HE on human liver cell lines Hepa-RG and LX-2 were evaluated using the 3-(4,5-Dimethylthiazol-2-yl)-2,5-Diphenyl Tetrazolium Bromide (MTT) assay, as shown in Figure 1. The data show that HE, administered at increasing concentrations (25, 50, 75, 100, and 150 µg/mL) for 48 h, induced a slight increase in cell viability, statistically significant at 25 µg/mL (Figure 1A) and the concentration of 150 µg/mL cell viability decreased significantly. In light of these data, in our study, 50, 75, and 100 µg/mL were considered for further analysis.

After treatment with the mixture of NAFLD Promoting Agents (NPAs), the cells were defined as Activated Cells (ACs). To evaluate the potential impact of NPAs on cell viability, the MTT assay was performed. A 48 h exposure resulted in a modest reduction in viability in both Hepa-RG (~10%) and LX-2 (~20%) cells (Figure 1B). Co-treatment with HE attenuated this effect, showing a significant improvement in cell viability at all tested HE concentrations, effectively restoring levels comparable to the untreated control (CTR) in Hepa-RG. Under the same conditions, LX2 cells showed a slight, non-significant, increase in cell viability (Figure 1B).

### 2.2. Intracellular Lipid Accumulation in Hepa-RG Cells Following NPAs Exposure: HE Attenuates Steatosis

**Figure 2:**
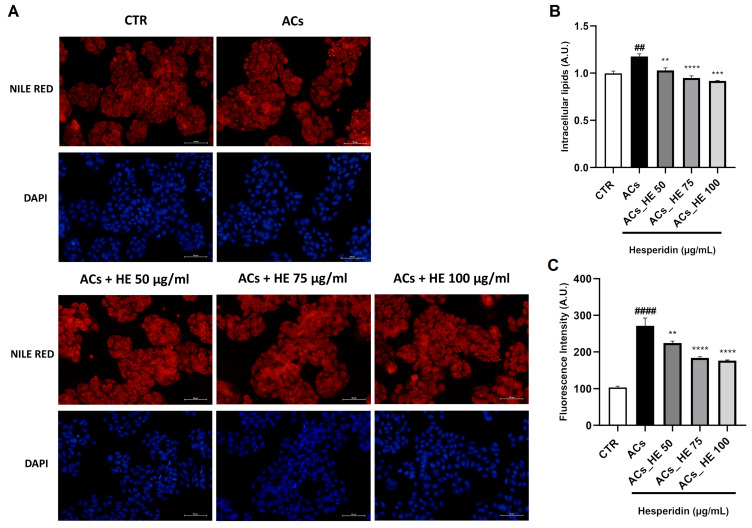
(**A**) Fluorescence of intracellular lipids by Nile Red and DAPI nucleus-staining dye. Effect of HE on lipid accumulation in activated Hepa-RG cells (ACs) treated with NPAs for 48 h (original magnification, ×40); representative images are shown. Scale bar is 50 μm. (**B**) Intracellular lipids after the quantification of Nile Red fluorescence using a microplate reader. Nile Red readings were normalized to the fluorescence of DAPI nucleus-staining dye, used as a control for cell number and proliferation. (**C**) The fluorescence intensity (number of pixels × mean intensity) of the Nile Red signal was quantified using ImageJ software 1.54g. Data were normalized to DAPI fluorescence to account for the cell number. Results are presented as mean ± SEM and counted as the total of 60 fluorescence microscope images (12 for each condition experimental) of three independent experiments (n = 3 for each condition). Statistical analyses: one-way ANOVA Dunnet’s post-hoc analysis; #: ACs compared to CTR, (## *p* < 0.01, #### *p* < 0.0001); *: HE compared to CTR, (** *p* < 0.01, *** *p* < 0.001, **** *p* < 0.0001). One-way ANOVA Dunnet’s post-hoc analysis to compare differences between CTR and activated cell groups (ACs).

To evaluate the pro-steatotic effect of NPAs, Hepa-RG cells were stained with the fluorescent dye Nile Red to detect neutral lipid droplets, while 4′,6-Diamidino-2-Phenylindole (DAPI) counterstaining enabled the normalization of lipid content to cell number. Fluorescent images in Figure 2A show that ACs exhibit a marked increase in intracellular lipid accumulation compared to control conditions. This result was further validated by the quantitative analysis of Nile Red fluorescence, revealing an increase of about 20% in lipid content in ACs compared to the control (Figure 2B). On the contrary, HE treatment for 48 h in ACs promoted a statistically significant reduction at all concentrations tested. Complementary image-based analysis with ImageJ software 1.54g, as illustrated in Figure 2C, indicated an even more pronounced almost three-fold (*p* < 0.0001) increase in lipid content in ACs, thus demonstrating the lipogenic capacity of NPA exposure and a statistically significant reduction after co-treatment with HE, thus confirming the anti-steatotic effect of the polyphenol [29].

### 2.3. Effect of HE on the Key Lipid Metabolism Mediators in Hepa-RG Cells

To elucidate the mechanism by which HE reduces hepatic lipid accumulation, we evaluated the protein expression of de novo fatty acid synthesis enzymes FASN and SCD1 by Western blotting.

**Figure 3:**
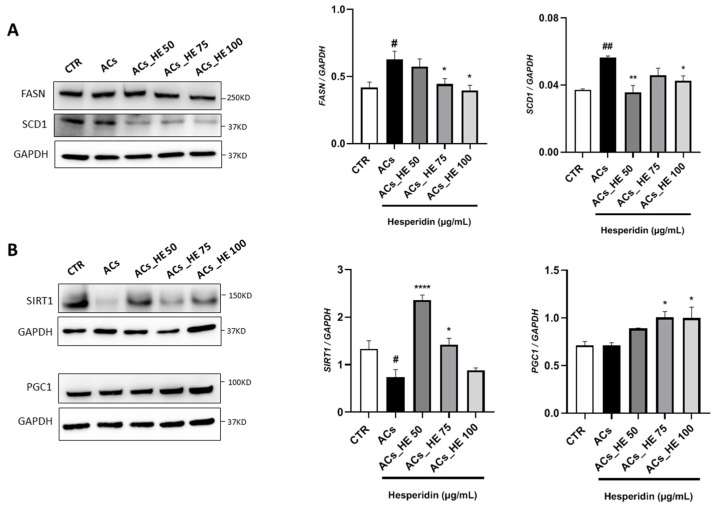
(**A**) Effect of HE on FASN and SCD1 protein expressions in activated Hepa-RG cells (ACs) was measured by Western blot, and GAPDH was used as a loading control. (**B**) The effect of HE on SIRT1 and PGC1 protein expressions in activated Hepa-RG cells (ACs) was measured by Western blot, and GAPDH was used as a loading control. Representative Western blot bands are shown. All data reported in each panel are expressed as the mean ± SEM from three independent experiments (n = 3 for each condition). Statistical analyses: one-way ANOVA Dunnet’s post-hoc analysis, *#*: ACs compared to CTR, (# *p* < 0.05, ## *p* < 0.01) and *: HE compared to CTR (* *p* < 0.05, ** *p* < 0.01, **** *p* < 0.001).

After activation with NPAs, Hepa-RG cells exhibited a statistically significant increase in FASN and SCD1 expression (Figure 3A). Subsequent treatment with HE resulted in a dose-dependent decrease in FASN protein levels, achieving significance at 75 µg/mL and 100 µg/mL. A similar reduction was also observed in SCD1 protein levels, with a statistically significant decrease evident starting from the lowest tested concentration of HE (Figure 3A). Based on the results described above, we decided to further investigate the action of HE in counteracting and improving NAFLD by examining its possible role in an additional fatty acid catabolism pathway, namely β-oxidation. Figure 3B shows significantly reduced SIRT1 levels in AC cells compared to CTR cells, while co-treatment with HE significantly increased expression at 50 µg/mL and 75 µg/mL compared to AC cells. Furthermore, we observed a statistically significant increase in PGC1α expression levels in ACs treated with HE at 75 µg/mL and 100 µg/mL compared to ACs.

### 2.4. HE Reduced the SCD1 Activity in Hepa-RG Cell Membranes

Table 1 shows the percentage values of fatty acids involved in SCD1 activity using a gas chromatography method. Interestingly, in Hepa-RG ACs a reduction in Saturated Fatty Acids (SFAs), such as stearic acid, and an increase in Monounsaturated Fatty Acids (MUFAs), such as oleic acid, compared with CTR were observed. In addition, both SCD1 and SCD1-C18 activity were increased compared with CTR. In contrast, these values change when ACs are treated with HE. In fact, following treatment with 50 µg/mL, SCD1 and SCD1-C18 decrease, as do MUFA levels, while SFA levels, palmitic acid, and stearic acid increase compared with ACs.

### 2.5. Effect of HE on the Gene and Protein Expression of Key Mediators Involved in Fibrogenic Processes in Activated LX-2 Cells

**Figure 4:**
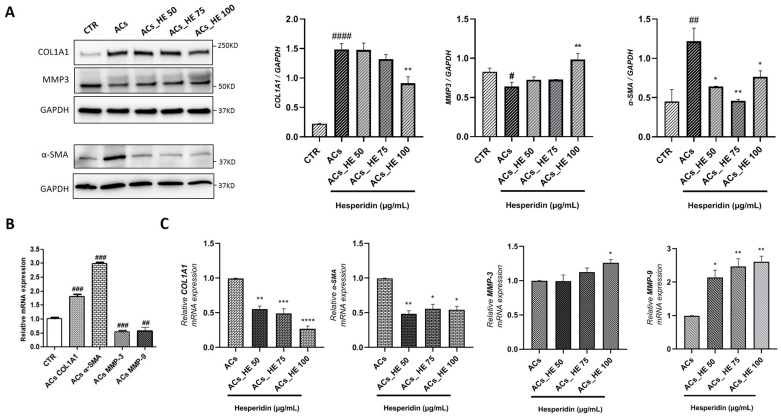
(**A**) The effect of HE of COL1A1, α-SMA, and MMP3 protein expressions in activated Hepa-RG cells (ACs) was measured by Western blot, and GAPDH was used as a loading control. Representative Western blot bands are shown. (**B**) COL1A1, α-SMA, MMP3, and MMP9 mRNA expression normalized by GAPDH in ACs compared with CTR. (**C**) COL1A1, α-SMA, MMP3, and MMP9 mRNA expression normalized by GAPDH in ACs treated with HE compared to ACs. All data reported in each panel are expressed as the mean ± SEM from three independent experiments (n = 3 for each condition). Statistical analyses: one-way ANOVA Dunnet’s post-hoc analysis, *: HE compared to CTR, (* *p* < 0.05, ** *p* < 0.01, *** *p* < 0.001, **** *p* < 0.0001) and #: ACs compared to CTR, (# *p* < 0.05, ## *p* < 0.01, ### *p* < 0.001, #### *p* < 0.0001).

The activation of HSC and the development of fibrosis are considered critical events in NAFLD [30]. Gene and protein expression levels of key fibrotic markers, including COL1A1, α-SMA, Matrix Metalloproteinase 3 (MMP3), and Matrix Metalloproteinase 9 (MMP9), were evaluated in ACs and ACs treated with HE. We observed that the protein expression levels of the profibrotic markers COL1A1 and α-SMA were significantly increased, while the expression of the antifibrotic marker MMP3 was markedly decreased in ACs compared to CTR. Interestingly, treatment with the HE significantly reduced COL1A1 protein expression at 100 µg/mL, while the α-SMA protein levels were decreased at all concentrations. In addition, HE significantly increased MMP3 protein levels at 100 µg/mL concentration compared to ACs (Figure 4A).

Protein analysis was further corroborated by the gene expression analysis of COL1A1, α-SMA, MMP3, and MMP9. As shown in Figure 4B ACs exhibited a significant upregulation of COL1A1 and α-SMA gene expression, along with a marked downregulation of MMP3 and MMP9 gene expression, compared to CTR cells. In contrast, HE leads to a significant reduction in the gene expression of COL1A1 and α-SMA, while MMP3 gene expression was elevated at HE 100 µg/mL, and MMP9 gene expression was significantly increased at all concentrations tested (Figure 4C).

## 3. Discussion

The pathogenesis of NAFLD is complex and appears to be influenced by dynamic interactions that are still poorly understood. A critical aspect in the development of predictive models for NAFLD and its more severe form, namely NASH, is the integration of relevant disease-inducing stimuli.

Hence, there is a significant need to develop NAFLD/NASH models capable of reproducing the key features of the disease aimed at evaluating therapeutic strategies for its prevention and treatment.

To date, plant-derived dietary supplements have been utilized in the management of liver diseases, including NAFLD [31]. It is important to emphasize that natural flavonoids, including HE, found in a variety of foods and beverages such as fruits, vegetables, tea, coffee, and wine, are known for their numerous beneficial effects on human health [32]. Several experimental models and emerging clinical trials have highlighted how these compounds can alleviate the damage caused by hepatic steatosis and improve liver function [15,33].

Based on our recent study showing that the daily consumption of Navelina oranges, which are particularly rich in HE, significantly reduces hepatic steatosis scores in patients with MAFLD, this study investigated, in vitro, the molecular mechanisms by which HE exerts its beneficial effects on the liver.

To the best of our knowledge, this is one of the few studies that develops a cellular model of hepatic steatosis using a cell line physiologically very similar to normal human hepatocytes. Hepa-RG and LX-2 cells exposed to NPAs mimic the development of NAFLD/NASH as it occurs in vivo. This treatment showed the key pathogenic features of NAFLD/NASH as enhanced steatosis, characterized by the upregulation of FASN and SCD1 and a concomitant reduction in β-oxidation-related pathways, as evidenced by a decreased expression of SIRT1 and PGC1α. SCD1 is a key enzyme in the conversion of SFAs into MUFAs, such as the transformation of palmitic acid into palmitoleic acid and stearic acid into oleic acid. An increase in SCD1 activity, as determined by lipidomic analysis in activated Hepa-RG cells, may be considered detrimental if associated with metabolic dysfunctions or alterations in lipid composition that compromise cellular health. The study of membrane lipid content through lipidomic analysis is innovative and it represents a valid method to evaluate cellular behavior during NAFLD development and treatment. Although MUFAs are generally considered beneficial for membrane fluidity, their excessive accumulation may disrupt the cellular lipid balance by reducing the proportion of SFAs, which are essential for maintaining membrane structural stability. An abnormal increase in MUFA content could result in overly “fluid” membranes, potentially impairing the function of membrane-bound proteins and receptors. Moreover, an increase in SCD1 activity may lead to an accumulation of MUFAs which, if not properly regulated, could contribute to the generation of Reactive Oxygen Species (ROS) and thereby promote oxidative stress [34,35,36,37]. Conversely, the reduction in SCD1 activity observed following HE treatment in activated hepatic cells appears to modulate fatty acid composition in a way that may confer protective effects on hepatic cell health. This reduction may promote the formation of more stable membranes, enhancing cellular resistance to oxidative stress and functional impairment. Additionally, increased membrane rigidity could offer protection against lipid peroxidation-related damage [38].

The results of our study highlighted a dual positive effect of HE in hepatocytes in countering hepatic steatosis: on one hand, HE was able to downregulate the expression of FASN and SCD1 proteins, which are essential for the regulation of de novo hepatic lipogenesis, the metabolic pathway responsible for converting carbohydrates into fatty acids [39,40]. On the other hand, HE promoted β-oxidation by upregulating the protein expression of SIRT1 and PGC1α. In summary, this led to an improvement in hepatic lipid accumulation, resulting in a consequent reduction in hepatic steatosis in our in vitro model. Some studies have shown that treatment with OA impairs the mitochondrial membrane potential, thereby disrupting mitochondrial function and the β-oxidation of fatty acids. As a result, intracellular fatty acids accumulate, which in turn promotes the generation of ROS, ultimately triggering hepatocellular apoptosis [41]. This cascade further triggers the activation of HSC, leading to fibrogenesis [42,43]. Consistent with these studies, we observed that NPAs (including OA and PA) caused a reduction in cell viability in Hepa-RG cells and in LX-2 cells compared to the control group. Interestingly, treatment with HE was able to counteract this NPA-induced cytotoxic effect, leading to a significant increase in cell viability at all tested concentrations in Hepa-RG cells and showing a positive trend in LX-2 cells. Moreover, the harmful effects of the NPA mixture clearly promote pro-fibrotic responses, as demonstrated by a marked increase in fibrosis markers such as COL1A1 and α-SMA, accompanied by a decrease in metalloproteinases, including MMP3 and MMP9. Notably, HE also induced a significant reduction in these markers.

In conclusion, our study provides compelling evidence that HE exerts a dual therapeutic effect by significantly attenuating hepatic steatosis and counteracting the cytotoxic and profibrotic effects induced by treatment with NPAs. These findings underscore the potential of HE as a promising agent for the prevention and treatment of NAFLD and its progressive form, NASH. Notably, an innovative aspect of our study is the application of lipidomic analysis to assess changes in the lipid composition of Hepa-RG cell membranes, particularly focusing on the modulation of SCD1 activity after HE treatment. This approach offers a valid method to monitor cellular behavior during disease progression and regression. Furthermore, the evaluation of HE’s protective effects on fibrotic processes was extended to LX-2 hepatic stellate cells, providing an integrated view of HE’s action against both steatosis and fibrosis.

The interpretation of our data and their extrapolation to clinical implications are yet too premature. Although in vitro models have inherent limitations, our findings strongly advocate for further in vivo studies to confirm the therapeutic potential of HE in NAFLD and NASH.

## 4. Materials and Methods

### 4.1. Cell Line and Culture Conditions

Hepa-RG was purchased from Thermo Fisher Scientific (Cat. No. HPRGC10) (Waltham, MA, USA) and cultivated according to the manufacturer’s protocols using a Hepatocyte Bullet Kit medium (HBM; Thermo Fisher Scientific, Waltham, MA, USA). The LX-2 was purchased from Millipore (Cat. No. SCC064) (Merck Life Science, Milan, Italy) and cultured in Dulbecco’s Modified Eagle’s Medium (DMEM; Thermo Fisher Scientific, Milan, Italy). Each medium was supplemented with 10% heat-inactivated Fetal Bovine Serum (FBS) and 1% antibiotic-antimycotic solution (10,000 U/mL penicillin, 10,000 μg/mL streptomycin, and 25 μg/mL Gibco Amphotericin B). Cells were expanded and maintained in the growth medium for two weeks at 37 °C in a humidified incubator with 5% CO_2_. For the NAFLD-inducing experiments, Hepa-RG and LX-2 cells were seeded in 6-well culture plates, cultured in a medium in the absence of FBS, and treated with a mixture prepared according to Rafiei et al. [6]. Specifically, the mixture contained insulin (10 nM, cod. I5500 Sigma-Aldrich, St. Louis, MO, USA), glucose (11 mM, cod. G8270 Sigma-Aldrich, St. Louis, MO, USA), Free Fatty Acids (100 µM oleic acid, cod. 031997.22 Sigma-Aldrich and 25 µM palmitic acid cod. 129702500 Sigma-Aldrich, St. Louis, MO, USA), 10 ng/mL lipopolysaccharides (LPS, cod. 44391 Sigma-Aldrich, St. Louis, MO, USA), and 3 ng/mL Transforming Growth Factor-Beta (TGF-β, cod. HY-P70543 MedChemExpress, Monmouth Junction, NJ, USA).

OA and PA were prepared as stock solutions at 120 mM and 75 mM, respectively, complexed with lipid-free Bovine Serum Albumin (BSA, cod. A7030 Sigma-Aldrich, St. Louis, MO, USA) to a final concentration of 1% BSA in the NPAs medium.

To establish the effect of HE, once the cells reached approximately 80% confluence, the culture medium was removed and replaced with a fresh medium containing HE (cod. H5254 Sigma-Aldrich, St. Louis, MO, USA) at three different concentrations (50, 75, and 100 µg/mL) for a 2 h pre-treatment. Subsequently, a specific NPA was added in the presence of HE treatment (50, 75, and 100 µg/mL) for 48 h. Control cells were treated with an equivalent volume/volume (*v*/*v*) concentration of the vehicle present at the highest treatment concentration of HE (100 µg/mL) Dimethyl Sulfoxide (DMSO; cod. D2438, Sigma Chemical, St. Louis, MO, USA). HE treatment concentrations (50, 75, and 100 µg/mL) were prepared from the 10,000 µg/mL concentration by diluting with the appropriate culture media.

### 4.2. Cell Viability Assay

The determination of the cell viability of Hepa-RG and LX-2 cells was evaluated by the MTT test (cod. M6494 Sigma-Aldrich, St. Louis, MO, USA). The test is based on the assessment of the activity of mitochondrial dehydrogenases, active only in living cells, which reduce MTT to formazan. After treatments of 48 h, MTT (0.5 mg/mL) solution was added to each well and the cells were incubated for 3 h at 37 °C, protected from the light, and the supernatant was removed. The formazan crystals were solubilized using acidic isopropanol (absolute isopropanol + 5% Hydrochloric Acid (HCl)). Then, the absorbance values at 570 nm were determined using a microplate reader (FLU Ostar Omega, BMG Labtech, Allmendgrün, Ortenberg Germany).

### 4.3. Qualitative Analysis of Intracellular FFA—Nile Red Staining

Intracellular lipid droplets were assessed using Nile Red staining (72485, Sigma-Aldrich, St. Louis, MO, USA). Briefly, Hepa-RG cells were seeded on coverslips in 48-well plates and allowed to adhere for 24 h. Subsequently, the cells were treated with the NPAs for 48 h, either in the presence or absence of HE. Following treatment, the cells were washed and fixed with 4% paraformaldehyde (P6148, Sigma-Aldrich, St. Louis, MO, USA) for 20 min at room temperature. After fixation, the cells were washed with PBS and incubated in Hank’s Balanced Salt Solution (HBSS) containing 1 μg/mL of Nile Red for 30 min at 37 °C in the dark. Nuclear staining was performed using DAPI (PureBlu™ DAPI Nuclear Staining Dye cat.135-1303, Bio-Rad Laboratories, San Francisco, CA, USA) at a concentration of 2 μg/mL in Phosphate-Buffered Saline (PBS). Subsequently, cells were gently washed twice with PBS, and the fluorescence was measured using a FLUOstar Omega microplates reader (BMG Labtech, Allmendgrün Ortenberg Germany), with excitation at 488 nm and emission at 585 nm. Fluorescence data were normalized to nuclear staining using DAPI to account for cell number and proliferation. The coverslips with the stained cells were mounted upside down on glass slides. In total, 60 fluorescence images (12 for each experimental condition) were acquired using an inverted fluorescence microscope (NIKON Eclipse Ti2, Tokyo, Japan; 40× objective) and an NIS fluorescence imaging system.

### 4.4. Western Blot Analysis

Hepa-RG and LX-2 cells were lysed in Radioimmunoprecipitation Assay Buffer (RIPA buffer) (Sigma-Aldrich, St. Louis, MO, USA) supplemented with the Halt Protease and Phosphatase Inhibitor (ThermoFisher Scientific, Waltham, MA, USA). The lysate was recovered and centrifuged at 14,000 rpm for 30 min at 4 °C, and the supernatant was collected and used for total protein quantification by a standard Bradford assay (Bio-Rad Laboratories, San Francisco, CA, USA). An equal amount of protein (30 μg) was separated in 4–15% Tris-glycine sodium dodecyl sulfate-polyacrylamide gel (Bio-Rad Laboratories, San Francisco, CA, USA). Membranes were incubated with gentle shaking, with the following primary antibodies: FASN (Rabbit mAb #94181, 1:800, Immunological Science, RM, Italy), SCD1 (Rabbit mAb #2438, 1:1000, Cell Signaling Technology, Danvers, MA, USA), SIRT1 (Rabbit, #VPA00294, 1:1000 Precision Ab, Bio-Rad Laboratories, San Francisco, CA, USA), PGC1α (Rabbit, #NBP1-04676, 2 μg/mL, Novus Biologicals a Biotechne Brand, Centennial, CO, USA), COL1A1 (Mouse mAb#66948, 1:1000, Cell Signaling Technology, Danvers, MA, USA), MMP3 (Rabbit mAb #14351, 1:1000, Cell Signaling Technology, Danvers, MA, USA), and α-SMA (Rabbit mAb #19245, 1:500, Cell Signaling Technology, Danvers, MA, USA).

The MMP3 antibody was diluted in 5% *w*/*v* no fat dry milk, 1X Tris-Buffered Saline (TBS), and 0.1% Tween^®^ 20. FASN, SCD1, COL1A1, and MMP9 antibodies were diluted in 5% *w*/*v* BSA, 1X TBS, and 0.1% Tween^®^ 20. Experiments were carried out in triplicate. After overnight incubation of primary antibodies, anti-rabbit or anti-mouse secondary antibody (1:5000 Bio-Rad Laboratories, San Francisco, CA, USA), diluted in 5% *w*/*v* no fat dry milk, 1X TBS, and 0.1% Tween^®^ 20, was incubated for one hour with gentle shaking. The chemiluminescence signal from proteins was revealed using Clarity Western ECL Substrate or Clarity Max Western Enhanced Chemiluminescence (ECL) Substrate (Bio-Rad Laboratories, San Francisco, CA, USA) and analyzed using the chemiluminescence detection system ChemiDoc XRS (Bio-Rad Laboratories, San Francisco, CA, USA). The relative density of the bands was calculated using the ImageLab software 5.2.1 and the proteins detected were normalized against the expression of the housekeeping gene GAPDH (Rabbiym Ab, #2118, 1:1000 Cell Signaling Technology, Danvers, MA, USA).

### 4.5. Lipidomic Analysis

Total lipids were extracted from the cell membranes of Hepa-RG CTR, ACs, and ACs HE with 50, 75, and 100 μg/mL HE using a modified Folch method [44]. Briefly, 450 μL of an acidified salt solution (H_2_SO_4_ 2 × 10^−4^ M, NaCl 0.1%) and 2250 mL chloroform–methanol (2:1) were added to 100 μL of cell pellet. The mixture was centrifuged at 3000 rpm for 20 min at 4 °C. The lower phase containing the fatty acids and chloroform was collected and evaporated with a centrifugal evaporator (Thermo Fisher Scientific, Waltham, MA, USA). The FA were transesterified into their respective Fatty Acid Methyl Esters (FAME) and analyzed by a Flame Ionization Detector (FID) gas chromatograph (AGILENT, Milan, Italy). A total of 1 μL of FAME was analyzed using a BPX70 0.25 μm capillary column (SGE Analytical Science, Milton Keynes, UK). Hydrogen was employed as the carrier gas at a constant flow rate of 3 mL·min^−1^. The injector and flame ionization detector were maintained at 250 °C. Fatty acid quantification was expressed as the relative percentage of total fatty acid content. Peaks were identified by comparing them to a standard mixture (Supelco 37-Component FAME Mix; Sigma-Aldrich, Milan, Italy).

Among the 37 FAs analyzed in cell membranes, special attention was paid to key representatives of the two major FA families: SFAs, particularly palmitic and stearic acids; and MUFAs, such as palmitoleic and oleic acids. In addition, the activity of the two SCD1 desaturase enzymes was calculated as the sum of the ratios of SCD1-C16 (palmitoleic/palmitoic acid) and SCD1-C18 (oleic/stearic acid).

### 4.6. Nucleic Acid Extraction and RT-qPCR/qPCR

Total RNA was extracted from Hepa-RG and LX-2 cells with the RNeasy Mini kit according to the manufacturer’s instructions (QUIAGENE, Hilden, Germany). RNA reverse transcription was performed using iScript Adv cDNA kit for RT-qPCR (Bio-Rad Laboratories, San Francisco, CA, USA). cDNA was then analyzed by qPCR with the SYBRGreenER™ qPCRSuperMix Universal (Thermo Fisher Scientific, Milan, Italy). RNA expression level was determined using the comparative cycle threshold (Ct) method, where the amount of target cDNA was normalized to housekeeping genes GAPDH cDNA. When cells were treated with HE, the amount of target cDNA was normalized to housekeeping genes and to the control cDNA (2^−ΔΔCt^). Primers used for the qPCR step are presented in Table 2.

### 4.7. Statistical Analysis

Data were expressed as the mean ± SEM and all experiments were performed in three independent experiments (n = 3). Data were analyzed using the GraphPad 8.0.1 software. One-way ANOVA corrected for multiple comparisons by Dunnet’s post-hoc analysis was performed to compare differences in lipidomic profiles and to evaluate the differences in protein expression between CTR and treated cell groups. Statistical significance was set at * *p* < 0.05, ** *p* < 0.01, *** *p* < 0.001, **** *p* < 0.0001.

## Figures and Tables

**Table 1:** Relative percentage of membrane fatty acid profile in Hepa-RG cell lines.

	CTR	ACs	ACs HE 50 μg/mL	ACs HE 75 μg/mL	ACs HE 100 μg/mL
Palmitic acid	22.11 ± 0.02	22.03 ± 0.10	23.64 ± 0.29 ***	24.72 ± 0.01 ***	23.65 ± 0.30 ***
Palmitoleic acid	0.77 ± 0.01	0.80 ± 0.02	0.60 ± 0.002	0.31 ± 0.08 ***	0.43 ± 0.10 ***
Stearic acid	15.32 ± 0.15	13.22 ± 0.21 ^##^	17.11 ± 0.36 ***	18.25 ± 0.05 ***	16.29 ± 0.60 ***
Oleic acid	31.96 ± 0.12	35.55 ± 0.07 ^###^	34.43 ± 0.37	35.76 ± 0.80	35.47 ± 0.10
SFAs	42.44 ± 0.18	39.88 ± 0.35 ^##^	45.47 ± 0.58 ***	46.73 ± 0.24 ***	44.31 ± 0.76 ***
MUFAs	40.62 ± 0.10	43.01 ± 0.17 ^##^	40.61 ± 0.47 **	41.38 ± 0.75	42.26 ± 0.08
SCD1-C16	0.035 ± 0.002	0.037 ± 0.002	0.027 ± 0.002	0.012 ± 0.003 ***	0.018 ± 0.005 **
SCD1-C18	2.09 ± 0.03	2.70 ± 0.05 ^###^	2.03 ± 0.06 ***	1.96 ± 0.05 ***	2.20 ± 0.08 ***
SCD1	2.13 ± 0.03	2.73 ± 0.05 ^###^	2.05 ± 0.07 ***	1.97 ± 0.05 ***	2.22 ± 0.08 ***

All values are expressed as the mean ± SEM of nine biologically independent samples (n = 9 for each condition). One-way ANOVA corrected for multiple comparisons by Dunnet’s post-hoc analysis; #: ACs compared to CTR (## *p* < 0.001; ### *p* < 0.0001); *: HE compared to CTR (** *p* < 0.001; *** *p* < 0.0001). Abb: SFAs, Saturated Fatty Acid; MUFAs, Monounsaturated Fatty Acid; SCD1, Stearoyl-CoA Desaturase-1; SCD1-C16 (palmitoleic/palmitoic acid); SCD1-C18 (oleic/stearic acid).

**Table 2:** Unique assay ID of the primers used to measure the expression genes of interest.

Gene	Unique Assay ID	Chromosome Location	Amplicon Length
*COL1A1*	qHsaCED0043248	17:48277174–48278779	113
*α-SMA*	qHsaCID0013300	10:90699374–90701011	78
*MMP3*	qHsaCID0006170	11:102711183–102712920	148
*MMP9*	qHsaCID0011597	20:44641165–44641948	82
*GAPDH*	qHsaCED0038674	12:6647267–6647413	117

## Data Availability

Data is available from the corresponding author upon reasonable request.

## Abbreviations

NAFLD	Non-alcoholic fatty liver disease
NASH	Non-alcoholic steatohepatitis
MAFLD	Metabolic Dysfunction-Associated Fatty Liver Disease
HE	Hesperidin
HCC	Hepatocellular Carcinoma
DNL	De Novo Lipogenesis
FFA	Free Fatty Acid
TG	Triglycerides
ACC	Acetyl-CoA Carboxylase
FASN	Fatty Acid Synthase
SCD1	Stearoyl-CoA Desaturase-1
PGC1α	Peroxisome Proliferator-Activated Receptor Gamma Coactivator 1 alpha
SIRT1	Silent Information Regulator 2 homolog 1
α-SMA	Alpha-Smooth Muscle Actin
COL1A1	Collagen Type 1 Alpha 1 Chain
COL1A2	Collagen Type 1 Alpha 2 Chain
GAPDH	Glyceraldehyde-3-Phosphate Dehydrogenase
ECM	Extracellular Matrix
SMAD 2/3	Small Mother Against decapentaplegic 2 and 3
HSC	Hepatic Stellate Cells
HepG2	Hepatocellular carcinoma cell line
LX-2	Human Hepatic Stellate Cell Line
Hepa-RG	Human Hepatoma Cell Line
CTR	Control
AML12	Alpha Mouse liver 12
MTT	3-(4,5-Dimethylthiazol-2-yl)-2,5-Diphenyl Tetrazolium Bromide
NPAs	NAFLD Promoting Agents
ACs	Activated Cells
DAPI	4′,6-Diamidino-2-Phenylindole
SFAs	Saturated Fatty Acids
MUFAs	Monounsaturated Fatty Acids
SCD1	Stearoyl-CoA Desaturase1
MMP3	Matrix Metalloproteinase 3
MMP9	Matrix Metalloproteinase 9
ROS	Reactive Oxygen Species
OA	Oleic Acid
PA	Palmitic Acid
FBS	Fetal Bovine Serum
LPS	Lipopolysaccharides
TGF-β	Transforming Growth Factor-Beta
BSA	Bovine Serum Albumin
DMSO	Dimethyl Sulfoxide
HCl	Hydrochloric Acid
PBS	Phosphate-Buffered Saline
HBSS	Hanks’ Balanced Salt Solution
RIPA	Radioimmunoprecipitation Assay Buffer
SDS-PAGE	Sodium Dodecyl Sulfate Polyacrylamide Gel Electrophoresis
TBS	Tris-Buffered Saline
TBS-T	Tris-Buffered Saline with Tween 20
ECL	Enhanced Chemiluminescence
DMEM	Dulbecco’s Modified Eagle’s Medium
HBM	Hepatocyte Bullet Kit medium
FA	Fatty Acids
FAME	Fatty Acid Methyl Esters
FID	Flame Ionization Detector
SEM	Standard Error of the Mean
